# Synchronous choroid plexus papilloma and Wilms tumor in a girl, disclosing a Li-Fraumeni syndrome

**DOI:** 10.1186/s13053-020-00158-7

**Published:** 2021-01-06

**Authors:** Ofelia Cruz, Victoria Caloretti, Hector Salvador, Veronica Celis, Vicente Santa-Maria, Andrés Morales La Madrid, Mariona Suñol, Patricia Puerta, Jordi Muchart, Lucas Krauel, Cinzia Lavarino

**Affiliations:** 1 Department of Pediatric Oncology, Hospital Sant Joan de Déu, Esplugues de Llobregat, Passeig Sant Joan de Deu 2, 08950 Barcelona, Spain; 2 Department of Pathology, Hospital Sant Joan de Déu, Esplugues de Llobregat, Barcelona, Spain; 3 Department of Neurosurgery, Hospital Sant Joan de Déu, Esplugues de Llobregat, Barcelona, Spain; 4 Department of Diagnostic Imaging, Hospital Sant Joan de Déu, Esplugues de Llobregat, Barcelona, Spain; 5 Department of Surgery, Hospital Sant Joan de Déu, Esplugues de Llobregat, Barcelona, Spain; 6 Laboratory of Molecular Oncology, Hospital Sant Joan de Déu, Esplugues de Llobregat, Barcelona, Spain

**Keywords:** Li-Fraumeni syndrome, TP53 mutation, Cancer predisposition syndrome, Synchronous neoplasia, Wilms tumor, Choroid plexus papilloma, Choroidal plexus tumors

## Abstract

**Background:**

Li-Fraumeni Syndrome (LFS) is a cancer predisposition syndrome characterized by the early-onset of multiple primary cancers which can occur at different moments (metachronous onset) or, more rarely, coincidentally (synchronous onset). Here we describe a previously unreported patient with presentation of synchronous Wilms tumor and Choroid plexus papilloma, leading to the diagnosis of a Li-Fraumeni Syndrome (LFS).

**Case presentation:**

A 6-year-old girl without previous complains presented with abdominal pain. Abdominal US and MRI showed a left renal tumor with subcapsular hematoma. Due to mild headaches, the diagnostic workup included a brain MRI that unexpectedly identified a large left parietal lobe tumor. Histopathological analysis determined the diagnosis of classic Wilms tumor and choroid-plexus papilloma (CPP), respectively. Both neoplasms showed intense nuclear p53 immunostaining associated with the pathogenic TP53 mutation c.844C > T (p.Arg282Trp). Our patient and her father shared the same heterozygous germline TP53 mutation, confirming the diagnosis of familiar Li-Fraumeni syndrome in the girl. The treatment was tailored to simultaneous tumor presentations.

**Conclusions:**

LFS has been associated with Choroid plexus carcinoma (CPC), but rarely with CPP as in our patient. That suggests that it may be advisable to consider the possibility of analyzing TP53 mutation, not only in all patients with CPC, but also in some patients with CPP, especially when histological or clinical evidences point out to perform this study. The dissimilar presentation of LFS among our patient’s father, not having so far any neoplasia diagnosed, while her daughter presented precociously with two simultaneous different tumors, could be related to possible effects of modifier genes on the underlying mutant p53 genotype.

**Supplementary Information:**

The online version contains supplementary material available at 10.1186/s13053-020-00158-7.

## Background

The presentation of two or more different neoplasias in the same patient is an unusual situation that suggests an underlying cancer predisposition condition. Li-Fraumeni Syndrome (LFS) is a cancer predisposition syndrome characterized by the early-onset of multiple primary cancers such as breast cancer, soft tissue and bone sarcomas, brain tumors and adrenal cortical carcinoma (ACC) [1]. The diagnosis of multiple cancers can occur at different moments (metachronous onset) or, more rarely, coincidentally (synchronous onset) [2]. Here we report a patient with synchronous Wilms tumor and Choroid plexus papilloma (CPP) leading to the diagnosis of a Li-Fraumeni Syndrome (LFS), situation that has not been described so far in the literature.

## Case presentation

**Fig. 1:**
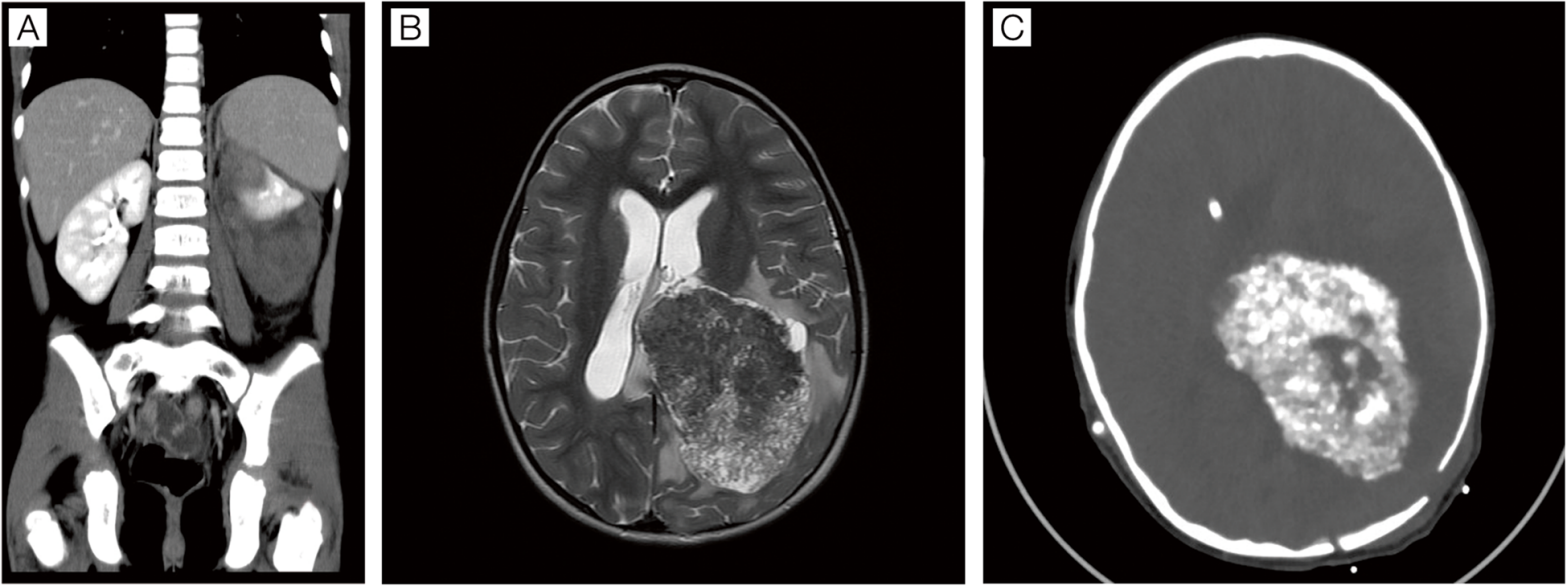
Radiological studies. Panel **a** Intravenous contrast-enhanced abdominal CT, coronal view: a solid, heterogeneous tumor in the upper pole of the left kidney is identified (upper, thin arrow), with an adjacent subcapsular perirenal hematoma (lower, thick arrow). The actual diameters were 36 mm × 28 mm × 27 mm in anteroposterior, transverse, and craniocaudal planes. Panel **b** Cerebral MRI, T2-weighted sequence, axial view. A solid supratentorial intraventricular tumor with left parietal lobe extension is shown. Cystic areas in the basal peripheral and medial aspects are identified .The tumor causes localized ventriculomegaly, vasogenic edema and midline shift. The actual diameters (tumor and cysts) were 81 × 59 × 75 mm in the anteroposterior, transverse, and craniocaudal planes. Panel **c** Non-enhanced Brain CT scan after shunt insertion. Axial reconstruction. A left parietal tumor with extensive calcification is shown. Ventricular catheter tip is in the right frontal ventricular horn

A 6-year-old girl patient presented to the ER (emergency room) with an intense abdominal pain accompanied by pallor, vomiting, sweating and hematuria. The abdominal ultrasound revealed the presence of a nodular lesion in the left kidney and local hemorrhage. Her medical history was unremarkable except for occasional self-limited episodes of mild headache. She had normal developmental milestones, a satisfactory school performance and brisk dancing skills. Some family members were diagnosed with carcinomas, all with adult-onset. At admission, her physical examination was remarkable for abdominal tenderness without any evident neurological deficits. The abdominal CT scan ruled out acute arterial bleeding and an MRI confirmed a tumor in the upper pole of the left kidney with extensive intracapsular hemorrhage, suggestive of a Wilms’ tumor (Fig. 1 panel a). Due to the episodes of headache, the initial workup was extended with a brain MRI that the presence of a fairly large left supratentorial tumor with high cellularity and vascularization, features suggestive of high-grade neoplasia (Fig. 1 panel b). There was no evidence of leptomeningeal, lung, or systemic dissemination. Deeper neurological examination revealed full right hemianopia, which had been detected to that moment.

**Fig. 2:**
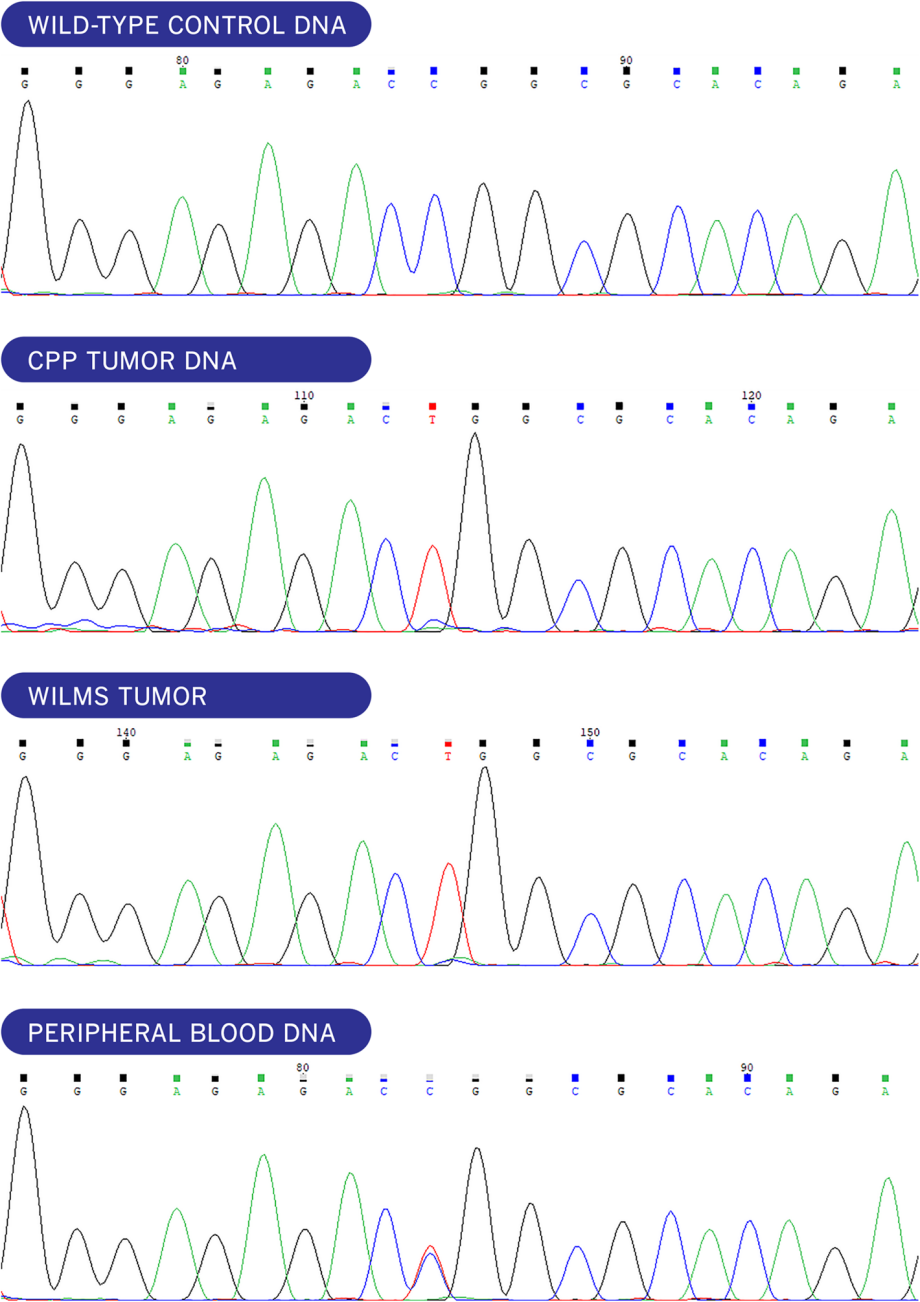
Findings from the molecular genetic analysis of TP53 gene in patient’s tissues. Sanger sequencing. From top to bottom traces show wild-type control DNA, patient’s CPP DNA, Wilms tumor DNA, both carrying the pathogenic TP53 mutation in homozygous status consistent with the absence of the wild type allele, and in the lower trace the heterozygous germline TP53 mutation. The TP53 missense mutation in exon 8 (c.844C > T; p.R282W; CGG > TGG) is marked in yellow

Since the renal hemorrhage improved spontaneously during the initial evaluation period, the cerebral tumor surgery was prioritized. The initial neurosurgery could only achieve a partial resection due to the high vascularity and extremely hard texture of the tumor, requiring intraoperative transfusional support. Postoperative hydrocephalus required a ventriculo-peritoneal shunt. The CT scan after the shunt procedure showed a highly calcified tumor (Fig. 1, panel c). The histological analysis identified a choroidal plexus tumor composed of well-differentiated papillary pattern covered by cells with nuclear pleomorphism and cytological atypia, but no evidence of solid pattern, mitotic figures or necrosis were identified (supplementary material). Intense p53 nuclear staining was identified by immunohistochemistry (IHC) in the near-totality of cells. The Ki67 stain was positive in less than 10% of cells. The histopathological diagnosis was plexus papilloma with atypical features. However, due to difficulty in evaluation of the grade of this tumor, an international review was requested which confirmed the diagnosis of a typical choroid plexus papilloma (CPP) (Dr. Martin Hasselblat, international CPT registry [3] also see acknowledgments-. Sanger sequencing of TP53 (NM_000546.6) identified in the CPP the missense variant c.844 C > T (p.Arg282Trp), previously reported as a pathogenic/likely pathogenic mutation (COSM10704; dbSNP variant rs28934574). The wild-type allele was absent, consistent with the loss of heterozygosity (Fig. 2). Germline TP53 mutation analysis was performed. Sanger sequencing revealed the presence of the c.844C > T mutation in heterozygous status in the patient’s peripheral blood. Informed consent signed by parents was obtained before germline testing. Our findings confirmed the suspected Li–Fraumeni syndrome as the cancer predisposition syndrome underlying the patient’s malignancies (Fig. 2). Genetic testing of the family disclosed that she shared with her healthy father the same TP53 mutation, while both, her mother and sister, had no deleterious variants.

After the first neurosurgery, the administration of a course of ICE chemotherapy (Ifosfamide-carboplatin-etoposide) [4] was given to facilitate a second look surgery, but also considering that would be useful for both the choroid plexus tumor and the likely concomitant Wilms tumor. After one ICE Course the MRI evaluation showed that the cerebral tumor was stable, whereas the renal tumor was slightly reduced and the pericapsular hematoma was completely resolved. Before the second neurosurgery a cerebral arteriography was performed without detecting any actively dependent vessels, therefore tumor embolization was not performed. In the second neurosurgery only partial resection could be accomplished because of hemodynamic instability due to hemorrhage. The histopathological analysis confirmed CPP with atypical features and prominent osseous and cartilaginous metaplasia. The residual tumor caused partial exclusion on the left lateral ventricle. An Ommaya-type reservoir with the catheter tip implanted into the excluded ventricle permitted withdrawal of CSF and resolved the symptoms of the segmental ventriculomegaly.

After confirming the stability of the cerebral tumor, complete resection was deferred in order to focus on the renal tumor. A radical left nephrectomy showed a Stage II Wilms’ tumor without anaplasia, but nuclear unrest was patent; p53 IHC staining was very strong and Sanger sequencing of TP53 demonstrated the presence of the same homozygous c.844C > T mutation identified in the CPP tumor (Fig. 2). Chromosomal analysis (CytoScan HD Array, ThermoFisher Scientific) of the Wilms’ tumor revealed a complex pattern of copy-number gains and losses affecting numerous chromosomes (Supplementary Figure C). Chromosome 17 displayed two chromosomal abnormalities, a long contiguous region of absence of heterozygosity encompassing the entire short arm of chromosome 17 associated with an extra copy of the entire chromosome (trisomy). The short arm of chromosome 17 displayed thus three homozygous, uniparental copies. More than 70% of the viable tumor consisted of blastema. Chemotherapy was tailored to the blastemal-type Wilms tumor and the persistent CPP, with alternating cycles of VAD chemotherapy (Vincristine / Actinomycin / Doxorubicin) [5] with ICE, for four courses. Although the role of adjuvant chemotherapy is controversial in CPP, the aim of the treatment was to facilitate the surgical resection [6]. At the end of chemotherapy the patient underwent a third intervention for the brain tumor and a complete resection was achieved with an uneventful postoperative course. The histology confirmed CPP.

At the time of this report, more than 2 years follow-up after diagnosis, the patient is in complete remission with no new tumors identified during the follow-up. So far, her father has regular follow-up examination and has not been diagnosed with any type of neoplasia.

## Discussion

Li-Fraumeni syndrome (LFS) is a cancer predisposition syndrome caused by heterozygous germline mutations in the TP53 tumor suppressor gene on chromosome 17p13.1 (LFS, OMIM# 151623) [7]. The mutation may be inherited from one of the parents with an autosomal dominant manner or may be due to a spontaneous (de novo) genetic mutation. TP53 is a key regulator of cell cycle checkpoints and apoptosis. Upon activation by cellular stress, particularly DNA damage, p53 binds DNA and induces the transcription of downstream genes involved in apoptosis, cell cycle arrest and DNA repair [8]. Somatic mutations in the TP53 gene are one of the most frequent alterations identified in sporadic human cancer.. The p.Arg282Trp mutation diagnosed in our patient involves a recognized “hot-spot” residue in p53’s DNA-binding domain, among the most common TP53 mutations in human cancers [9], and has been previously described associated with early onset of familial cancers [10].

The spectrum of LFS neoplasias includes typically premenopausal breast cancers, soft tissue sarcomas, brain tumors and adrenocortical carcinomas (ACC) [1, 2, 11, 12]. Unlike in adults with LFS, children can also develop typical “pediatric” embryonal tumors [11, 13, 14]. The most frequent neoplasias associated to LFS in pediatric age are osteosarcoma, rhabdomyosarcoma, low hypodiploid leukemia [14], ACC, choroidal plexus carcinomas (CPC), SHH medulloblastomas [15], likewise Wilms tumor or neuroblastoma have also been reported. Choroid plexus tumors represent only 3% of pediatric brain tumors. There are three histologic variants: papillomas, atypical papillomas, and carcinomas. The CPPs outnumber CPCs by a ratio of at least 5: 1 [3, 16]. While the association of CPC with the Li-Fraumeni Syndrome is well known, the association with CPP is very rare [17, 18]. Here, we report a patient with synchronous Wilms tumor and choroid plexus papilloma (CPP). It is of interest that LFS has been associated with CPC, but not with CPP as in our patient. Based on these findings, it may be advisable to consider the possibility of analyzing TP53 mutation, not only in all patients with CPC, but also in some patients with CPP, especially when histological or clinical evidences point out to perform this study .

The management of neoplasias in the context of a LFS should be planned with a curative intent although the prognosis in some instances can be worse [14, 15]. Local therapy with surgery without irradiation stands as a mainstay element of therapy because these patients have an increased risk of developing secondary neoplasm after radiation therapy, therefore the recommendation is to avoid it if possible, especially when the tumor is likely to be locally controlled by surgery only [19–21]. Synchronous neoplasia in our case added diverse challenges to adjust the chronology of surgeries and the chemotherapeutic schema. Fortunately, according to histological or staging features both tumors were not candidate to radiation therapy. Moreover, as soon as the LFS was diagnosed in our patient, the use of CT scans and radiologic ionizing procedures was limited to prevent ionizing damage, e.g., performing one short MRI sequence in case of suspicion of shunt dysfunction instead of CT.

The diagnosis of LFS, with constitutional TP53 mutation can be suggested either by the characteristics of the first tumor, the identification of a second tumor as in our case, or else by performing familial genetic studies after an index case is diagnosed. Germline TP53 mutation testing recommendation have been summarized in the Chompret Criteria updated as the following: 1) After familial presentation of a proband with a LFS spectrum tumor prior to age 46 years and at least one first- or second-degree relative with a LFS tumor before the age of 56 years or with multiple tumors, 2) In the case of multiple malignancies and 3) In rare tumors as ACC, choroid plexus carcinoma or embryonal anaplastic subtype rhabdomyosarcoma or 4) Breast cancer before age 31 years [22].

The evaluation of TP53 gene status has important clinical implications for surveillance due to a high risk of developing cancer during lifetime. The risk of developing tumors is as high as 70% and the cumulative probability of a second cancer is 57% (± 10%) at 30 years. The use of a complete clinical surveillance protocol with laboratory tests and imaging such as whole-body MRI has allowed the early detection of asymptomatic tumors in TP53 mutations carriers, leading to an improved long-term survival [20–23].

Although our patient’s father harbored the same pathogenic germline mutation (p.Arg282Trp), he has not developed so far any neoplasia, while her daughter presented with two simultaneous tumors at early age of onset.. The p.Arg282Trp mutation has been reported as a hotspot variant at both germline and somatic levels associated with poorer prognosis as compared to other pathogenic missense variants [24]. Recent studies have described a potential gain of function effect of the p.Arg282Trp mutant, however further studies are needed to define the mechanisms through which this mutant protein affects the p53 signaling pathway. However, the heterogeneous and complex phenotype of LFS observed amongst affected family members is not readily explained by the unique identification of germline TP53 mutations; this variability in age of onset and tumor histotypes among affected individuals has been related to possible effects of modifier genes on the underlying mutant p53 genotype [10, 11, 25]. And also to a genetic anticipation with late cancer onset [26]. Furthermore, diverse studies have revealed that p53 mutations are biologically distinct. The same germline TP53 mutation may have different functional consequences in different tissue types. Alongside with the TP53 mutation, the order in which additional genetic alterations occur may collaboratively contribute and impact the timing and development of tumors [27].

## Conclusions

We report a new familial LFM patient with an unreported synchronous Wilms tumor and Choroid plexus papilloma. This is remarkable as that LFS has been associated with Choroid plexus carcinoma (CPC), but rarely with CPP as in our patient. That suggests that it may be advisable to consider the possibility of analyzing TP53 mutation, not only in all patients with CPC, but also in some patients with CPP, especially when histological or clinical evidences point out to perform this study may be analyzed not only in CPC, but also in CPP. On the other hand our patient’s father, harboring the same pathogenic germline mutation, has not developed so far any neoplasia, while her daughter presented with two simultaneous tumors at early age of onset. The heterogeneous phenotype of LFS observed amongst affected family members could be related to possible effects of modifier genes on the underlying mutant p53 genotype, or to a genetic anticipation, and this could explain the phenotypical differences between our patient’s father and her daughter.

## Supplementary Information

**Additional file 1.**

## Data Availability

Most data generated or analyzed during this study are included in this published article and its supplementary information files. Complete datasets generated and/or analyzed are not publicly available due to individual privacy could be compromised, but are available from the corresponding author on reasonable request.

## Abbreviations

ACC: Adrenocortical carcinoma
CPC: Choroid plexus carcinoma
CPP: Choroid plexus papilloma
CPT registry: Choroid plexus tumors registry
CT: Computerized tomography
CSF: Cerebrospinal fluid
ER: Emergency room
ICE: Ifosfamide Carboplatin Etoposide
IHC: Immunohistochemistry
LFS: Li Fraumeni Syndrome
MRI: Magnetic resonance imaging
VAD: Vincristine-Actinomycin-Doxorubicin
US: Ultrasound
WT: Wilms Tumor
