# Supplementary material for: Synchronous choroid plexus papilloma and Wilms tumor in a girl, disclosing a Li-Fraumeni syndrome
Source: Hered Cancer Clin Pract. 2021 Jan 6;19:1. doi: 10.1186/s13053-020-00158-7 (PMC7789227; doi:10.1186/s13053-020-00158-7)

SUPPLEMENTARY MATERIAL

The supplementary material includes three categories of figures:

1. Pedigree of this family
2. Histological images
3. Chromosomal analysis of Wilms Tumor.

FIGURE A: THE PEDIGREE OF THIS FAMILY.

Symbols with a diagonal line = deceased subjects; squares = male; circles = female; arrow = the proband. Information includes tumor type and tumor onset age.


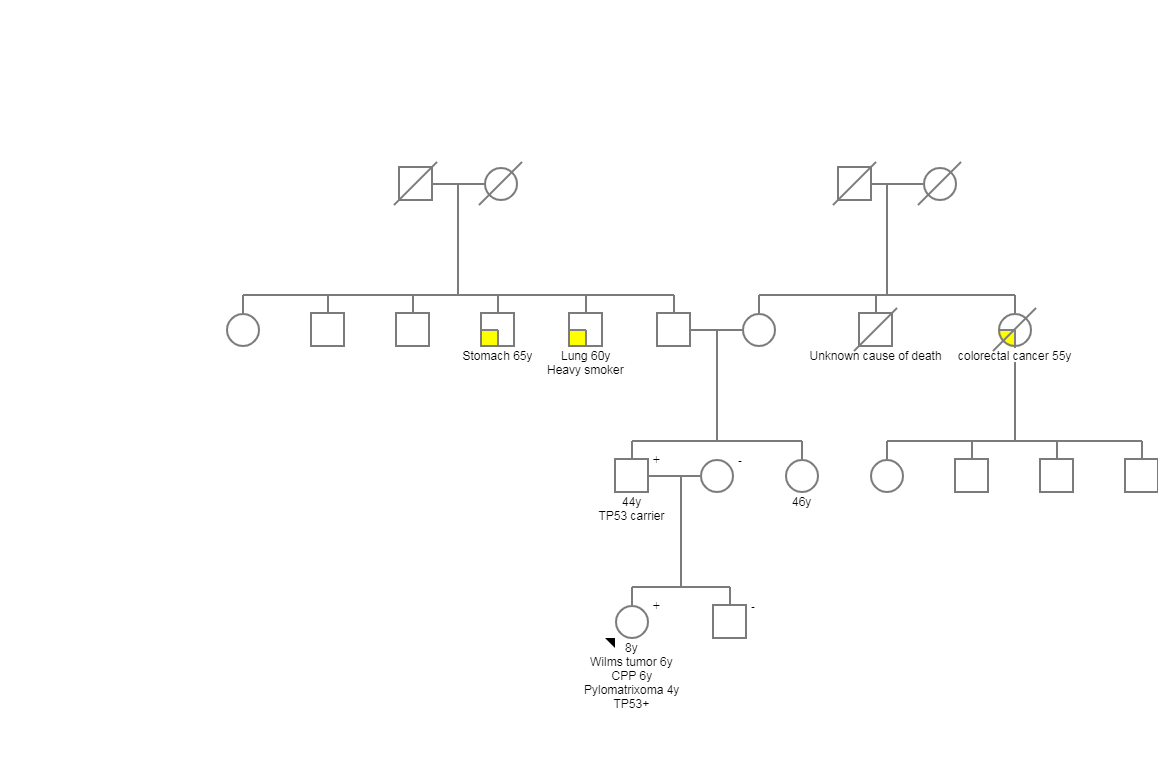


B. HISTOLOGICAL IMAGES: Photomicrographs of the choroid plexus tumor and of the synchronous Wilms tumor are described (Figures B1-B5).

Supplementary Figure B1: Photomicrograph of the choroid plexus tumor depicting osseous-cartilaginous metaplasia. Hematoxylin and eosin.


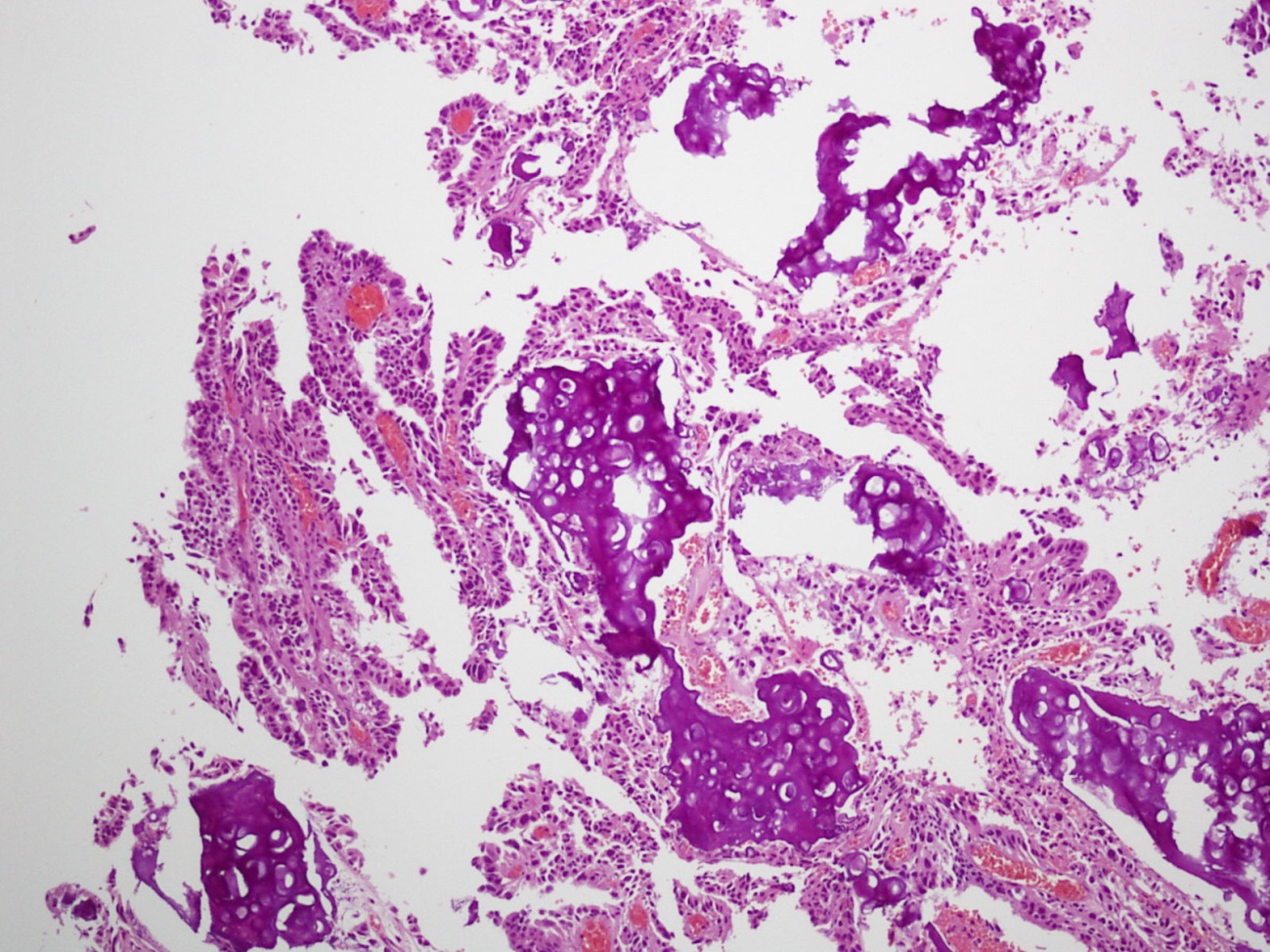


Supplementary Figure B2: Photomicrograph of the choroid plexus tumor depicting papillary structures and abundant calcifications. Hematoxylin and eosin.


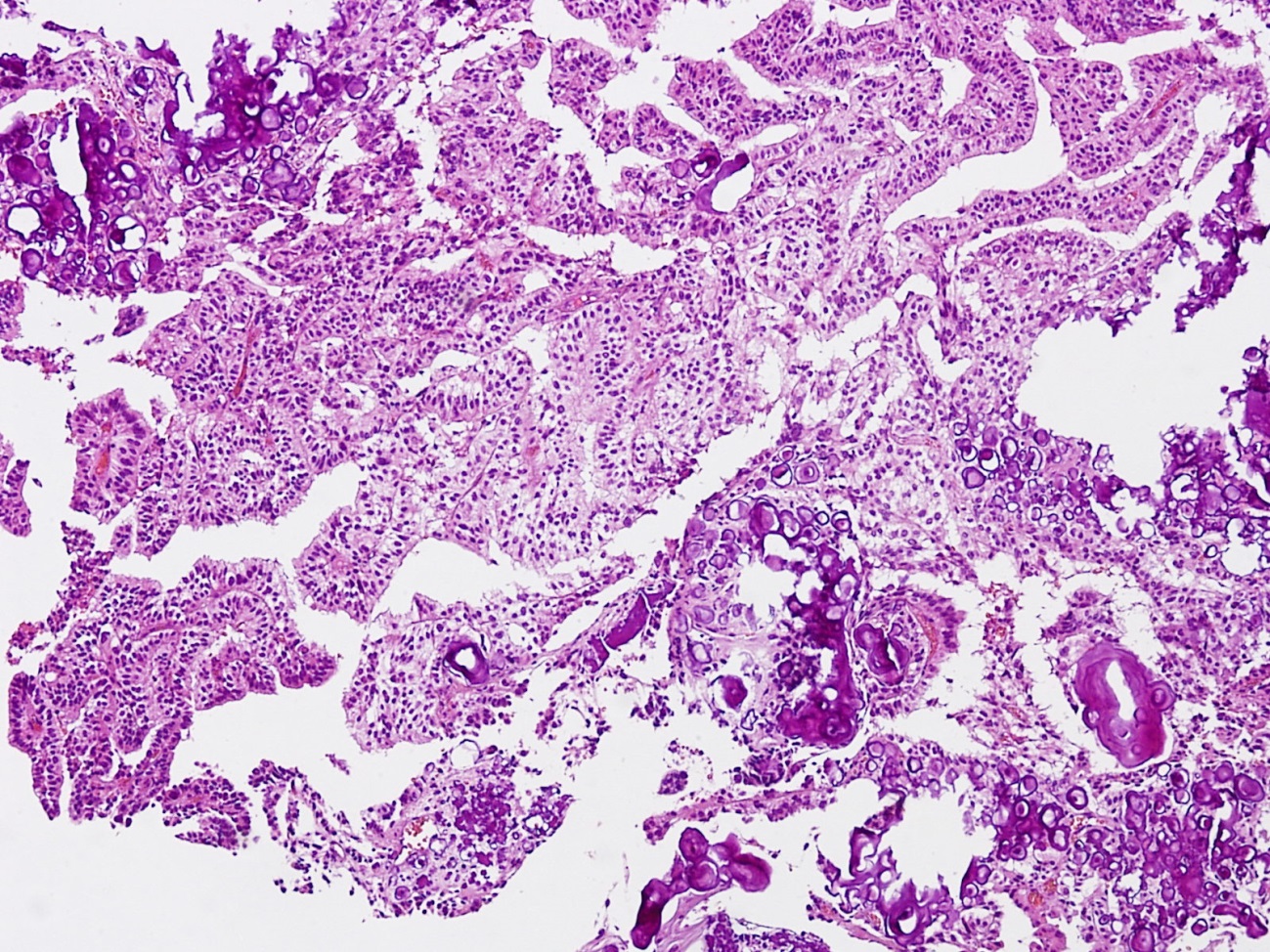


Supplementary Figure B3: Photomicrograph of the Wilms tumor depicting the blastema component. Hematoxylin and eosin.


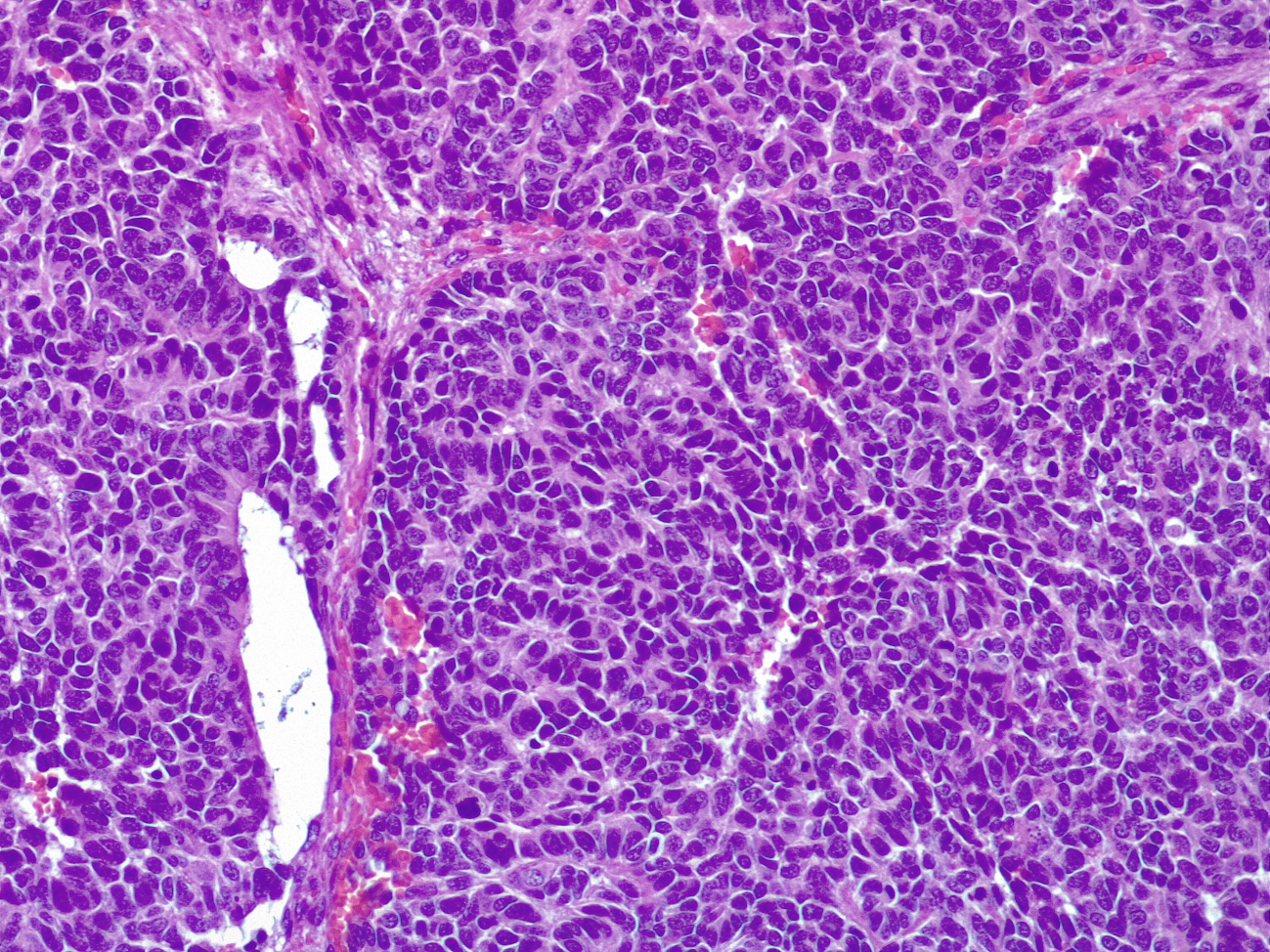


Supplementary Figure B4. Photomicrographs of the Wilms tumor depicting epithelial and stromal component. Hematoxylin and eosin.


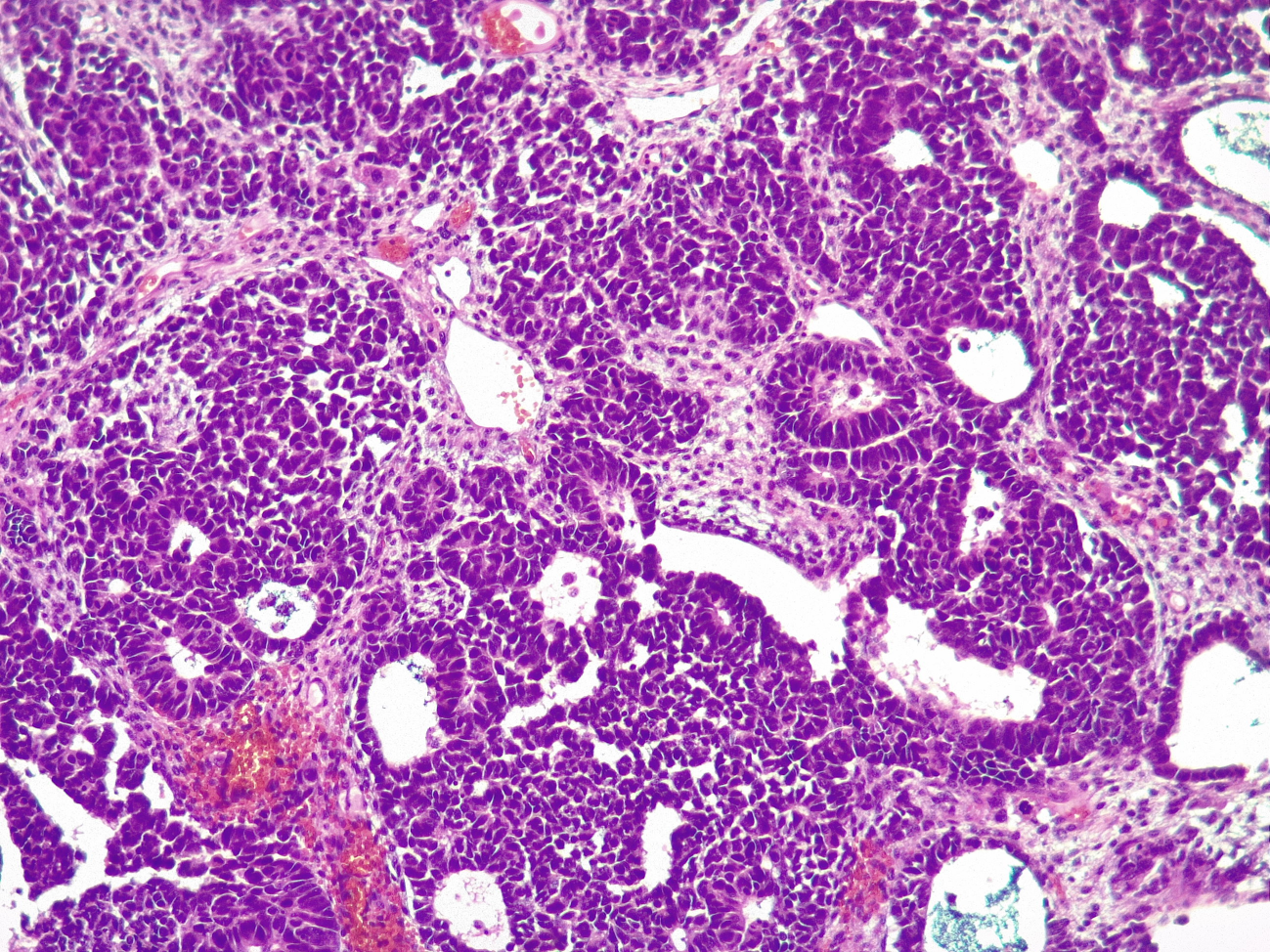


Supplementary Figure B5. Photomicrograph pf the Wilms tumor depicting undifferentiated cells without mitosis, pleomorphism or atypical mitosis. Hematoxylin and eosin.


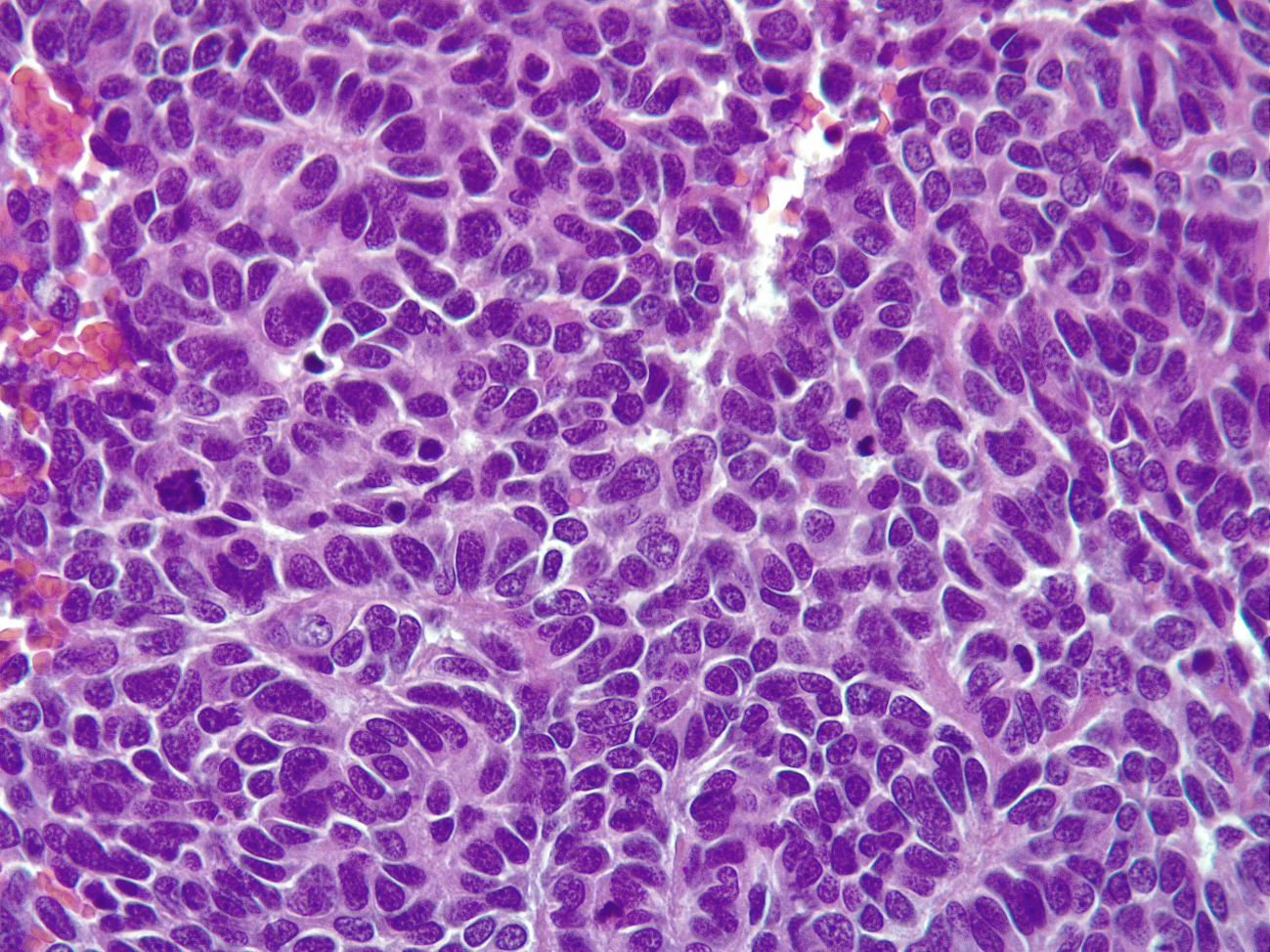


Supplementary Figure C. Chromosomal analysis.

Whole genome view of the chromosomal profile of the Wilms’ tumor displaying numerical and structural gains and losses affecting numerous chromosomes. Each colored column represents a different chromosome.


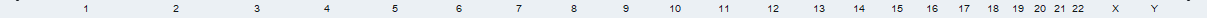

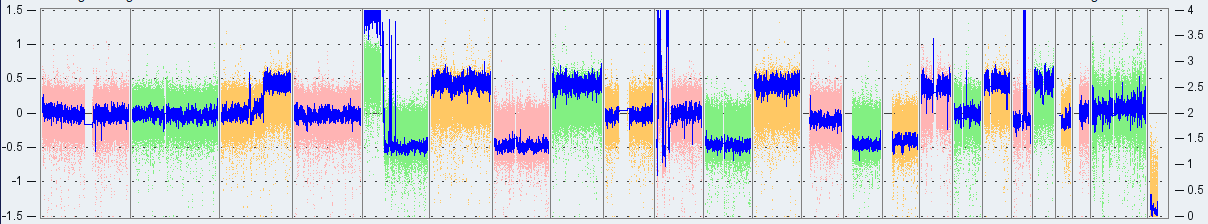

Supplement: Supplementary file 1 — Additional file 1. [file 13053_2020_158_MOESM1_ESM.docx]
